# The Contribution of Social Networks to the Health and Self-Management of Patients with Long-Term Conditions: A Longitudinal Study

**DOI:** 10.1371/journal.pone.0098340

**Published:** 2014-06-02

**Authors:** David Reeves, Christian Blickem, Ivaylo Vassilev, Helen Brooks, Anne Kennedy, Gerry Richardson, Anne Rogers

**Affiliations:** 1 National Institute for Health Research Collaboration for Leadership in Applied Health Research (NIHR CLAHRC) Greater Manchester, Centre for Primary Care, Institute of Population Health, University of Manchester, Manchester, United Kingdom; 2 National Institute for Health Research Collaboration for Leadership in Applied Health Research (NIHR CLAHRC) Wessex, Faculty of Health Sciences, University of Southampton, Southampton, United Kingdom; 3 School of Nursing, Midwifery and Social Work, University of Manchester, Manchester, United Kingdom; 4 Centre for Health Economics and NIHR Research Design Service for Yorkshire and the Humber, University of York, York, United Kingdom; University of St Andrews, United Kingdom

## Abstract

Evidence for the effectiveness of patient education programmes in changing individual self-management behaviour is equivocal. More distal elements of personal social relationships and the availability of social capital at the community level may be key to the mobilisation of resources needed for long-term condition self-management to be effective.

**Aim:**

To determine how the social networks of people with long-term conditions (diabetes and heart disease) are associated with health-related outcomes and changes in outcomes over time.

**Methods:**

Patients with chronic heart disease (CHD) or diabetes (n = 300) randomly selected from the disease registers of 19 GP practices in the North West of England. Data on personal social networks collected using a postal questionnaire, alongside face-to-face interviewing. Follow-up at 12 months via postal questionnaire using a self-report grid for network members identified at baseline.

**Analysis:**

Multiple regression analysis of relationships between health status, self-management and health-economics outcomes, and characteristics of patients' social networks.

**Results:**

Findings indicated that: (1) social involvement with a wider variety of people and groups supports personal self-management and physical and mental well-being; (2) support work undertaken by personal networks expands in accordance with health needs helping people to cope with their condition; (3) network support substitutes for formal care and can produce substantial saving in traditional health service utilisation costs. Health service costs were significantly (p<0.01) reduced for patients receiving greater levels of illness work through their networks.

**Conclusions:**

Support for self-management which achieves desirable policy outcomes should be construed less as an individualised set of actions and behaviour and more as a social network phenomenon. This study shows the need for a greater focus on harnessing and sustaining the capacity of networks and the importance of social involvement with community groups and resources for producing a more desirable and cost-effective way of supporting long term illness management.

## Introduction

Strategies for self-management focused on increasing patients' self-efficacy are often a key element of health policy for managing long term conditions. Patients taking on more responsibility for their health behaviours together with guided support and training has been viewed as a means of improving health outcomes and reducing the costs of health service utilisation[1]–[3]. However, this emphasis may not take advantage of the whole range of sources of support and the benefits to be gained from being linked into a wider set of community and social networks. In response to equivocal evidence of the effectiveness of patient education programmes designed to change individual behaviour[4] it has been suggested that more distal elements related to social relationships and the availability of social capital at the community level may be key to the mobilisation of resources needed to take self-management action [5].

Longitudinal studies of smoking, obesity, happiness, alcohol and drug use, have shown how social networks influence the genesis and spread of health related phenomena [6]–[10]. There are also known relationships between personal attributes associated with social networking such as altruism and volunteering, and health and well-being outcomes, particularly in older adults [11]–[15]. Social networks and the associated availability of social capital are also relevant for understanding flows of trust, reciprocity and social participation that underpin collective action and mutual support [16]. Low stocks of social capital, both at the community and individual levels, have been consistently shown to be strongly associated with poorer health outcomes [16], [17].

Social connectedness is important for social support and health, but more significant than the quantity of social relations is the perceived quality of these relationships [18]. Also, different types of relationships provide different kinds of support, thus a variety of types of ties are required to ensure stable and adaptable support [15]. A number of studies have reported on the interrelationships between the roles that people play in their networks and health, and how these change over a person's life-course (eg [19]). We have recently extended the social networks approach to consider the role that personal networks play in the lives of people with chronic health problems, not just concerning support for illness management, but also and equally importantly, the everyday practical and emotional challenges that living with a long-term condition entails [20], [21].

A social network perspective on condition management re-orientates the focus away from an individual's personal self-management actions to allow broader consideration of all the resources available to help support someone with a long-term illness. Shaw and Dorling [22] in an analysis based on the 2001 census, found that family and friends typically provide a considerable amount of care, with an average of one person proving 50 or more hours of unpaid care for every 3–4 people with a long-term condition. The authors also describe a strong “positive care” law at the district level with amounts of informal care increasing in direct proportion to degree of health need, in contrast to the inverse care law that dominates the geography of formal care services. However, the nature of the relationship between informal care and self-management support through a network, and utilisation of formal sources of healthcare is unclear, in particular whether the former substitutes for (and therefore reduces), or simply complements (and therefore does not reduce), the latter. The evidence for either relationship is inconclusive, and may well vary between different forms of formal care, such as community, outpatient, hospital, and national health system [23]–[26].

Social networks are also dynamic entities. Changes in a network can have a significant impact on the availability and use of resources in open or domestic settings where most support for people with long-term conditions takes place. Over time members of a network may move away, become ill themselves or die and resources may become less or more easily available [17], [19]. Close network members may be affected by the illness of the person they care for and their approach to the care given may change [27]. The negative aspects of illness have also been identified as producing relationship dynamics that can lead to social network attrition [27].

In this study we set out to determine whether and how the social networks of people with long-term conditions (specifically diabetes and heart disease) are associated with health-related outcomes and with changes in outcomes over time. We pay specific attention to the ways in which patient health and self-management are related to levels of social participation, characteristics of the members of the networks, and to the support received from these members, including whether the “positive care law” applies in this context, and to the nature of the relationship between informal care through the network and the use of formal health services.

## Methods

### Ethics Statement

All participants gave informed written consent to take part in the study. Ethical approval was obtained from the Greater Manchester Research Ethics Committee in February 2010 (ref: 10/H1008/1). All participants received £20 in gift vouchers as a compensation for their time and effort.

### Design and Sample Characteristics

The full details of the study design and sampling frame are given in our previous publications [5], [20], [21]. Patients with chronic heart disease (CHD) or diabetes were randomly selected from the disease registers of 19 consenting GP practices located predominantly in economically deprived areas of Greater Manchester in the North West of England and invited into the study. Baseline data was collected using a postal questionnaire, alongside face-to-face interviewing to collect details of personal social networks. We designed the study to have 90% power to detect a moderately low correlation between any pair of explanatory and outcome variables of 0.2, for which a total sample of 260 patients was required. Anticipating a loss-to-follow-up rate of around 15% (based on our previous studies with this population), we aimed to recruit 300 patients at baseline.

Invitation letters were sent out from practices but patient response was low, so to increase recruitment practice staff made telephone contact with invited patients to explain the study and answer questions. This led to a significant increase in recruitment. A total of 2,001 invitation letters were sent, in successive waves, until 300 patients had been consented and completed both the postal questionnaire and the interview (15% response rate). The sample was not intended to be representative as our aim was to reach a highly deprived population.

To identify the members of each participant's network we used the “name generator” approach; a common and validated method for identifying personal social networks [28]. Participants were asked to map social network members on a diagram of three concentric circles [18], placing members regarded as most important in relation to managing their condition in the central circle, less important members in the middle circle, and less important still in the outer circle. Participants could place as many network members as they wanted, of any type of relationship they considered relevant (e.g. family, friends, medical professionals, pets, groups, services). The face-to-face interviews also allowed - compared to a postal questionnaire - additional but initially overlooked network members to become visible during the interview and for detailed information to be collected about key attributes of each network member and their contribution to different illness-related activities.

Follow-up took place 12 months after baseline data collection. Data collection was via a postal questionnaire. To collect social network data at follow-up, a self-report grid was used that listed, for each participant, all the network members they identified at baseline for each of which the participant (i) indicated whether the member was still part of their network and (ii) rated the help received in each of three domains (managing their long-term condition; day-to-day tasks; emotional well-being) on a 1-5 scale from no help up to a lot of help. Participants were also asked to list and rate any new members of their network. The implications for the study of collecting network data in a different manner at follow-up are discussed later in the paper.

### Measures Used in the Study: Health Outcomes

The measures used in this study include data on patient demographics and social networks some of which has appeared in other publications from the study [5], [20], but which in this paper are related to a range of health-related outcome measures that have not been reported previously. For the purposes of presentation we have pragmatically divided the latter into two groups: health outcomes and health-economics outcomes. Health outcomes included two measures related to patients' abilities to self-manage their conditions plus measures of physical and emotional health status.

#### Self-management

To assess a patient's ability to self-manage their condition, we used the Health Education Impact Questionnaire (HEIQ). The HEIQ is a validated instrument originally designed for the evaluation of patient education and self-management interventions [29]. We used the Skill and Technique Acquisition (five items), and Self-monitoring and Insight (seven items) subscales of the full HEIQ as being the most relevant to self-management external to health service organisations. Both subscales had high in-sample internal consistency (Cronbach's alpha's of 0.86 and 0.81 respectively). They correlated highly (r = 0.65) and in view of this we computed and analysed each patient's average score across the two subscales.

#### Healthy behaviours

To measure the extent to which patients engaged in behaviours supportive of health we used the Summary of Diabetes Self-Care Activities scale (SDSCA). The SDSCA has been widely validated, with both English and other populations [30], [31], [32]. To produce comparable scores for CHD and diabetes patients we excluded items related to checking feet and blood sugar. The remaining seven items are dietary, exercise and smoking behaviours generally recommended for both diabetes and CHD patients (eg. following an eating plan; avoiding high-fat foods; regular exercise; not smoking). The in-sample internal consistency coefficient (Cronbach's alpha) for these seven items was an acceptable 0.65 [33]. The score represents the average number of days per week (out of 7) a participant followed healthy behaviours.

#### Physical health

We used the Short-form 12 (SF12) as a measure of physical health. The SF12 is one of the most well-validated and widely-used health status instruments [34] and can be analysed to obtain both physical and mental component scores. However, the standard item weights used to compute these are based on an assumption of independence between the two scores. There is considerable evidence that physical and mental health are in fact strongly related and that scores derived under this assumption are distorted for substantial numbers of patients [35]. We therefore used structural equation modelling (SEM) to examine the factor structure of the physical and mental components within the study sample, and found a high correlation, r = 0.83. In view of this, we decided to analyse only the physical component score (derived using item weights from the SEM) and exclude the mental component.

#### Emotional well-being

We computed an emotional well-being score for each patient by combining responses across two items: ‘Taking all things together, how happy would you say you are?’ and ‘All things considered, how satisfied are you with your life as a whole nowadays?’ The items were taken from the European Social Survey 2010 [36] and each was rated on a scale of zero (extremely unhappy/dissatisfied) to 10 (extremely happy/satisfied). The two items had an internal consistency coefficient (Spearman-Brown reliability as just two items [37]) of 0.89. We added the scores and rescaled to a range of 0 to 100.

### Health Economics Outcomes

We constructed two measures for each patient relating to a health economics assessment: quality-adjusted life years (QALYs); and health service costs. Responses to the SF12 at Time 1 and Time 2 were transformed into SF6D states and corresponding “utility” values using published algorithms [38]. A QALY value for the 12 month period was then calculated by following the area-under-the-curve (AUC) method [39].

Data on patient use of primary and secondary care services in the 6 months prior to Time 1 and Time 2 were collected in the patient questionnaires. Primary care resource use consisted of GP visits (at surgery, at patients' home and other) and practice nurse visits; secondary care use consisted of visits to A&E units, outpatient or day hospital attendances, and number of overnight stays in hospital wards. There can be trade-offs between primary and secondary care use and since our primary question was whether personal networks impact on resource use in general, we combined the two for analysis. Total service use costs were estimated by applying unit cost estimates to the amount of each type of resource use and summing.

### Explanatory Variables

#### Patient characteristics

Socio-demographic characteristics of respondents included age, gender, ethnicity, residential deprivation (the area Index of Multiple Deprivation 2007 [40]), occupational class, highest qualification, and income. As proxies for disease burden we used main disease condition (diabetes, CHD, or both) and total number of long-term conditions.

#### Network member characteristics

The characteristics collected about each social network member and the measures we constructed from these were based on factors found to be relevant to outcomes in previous studies of social networks and health [18], [41], [42], [43]. Characteristics included type of relationship, distance, amount of contact and form of contact and from these we constructed network-level measures of: the number of ‘proximate children' (children living in the same home or within a 5-minute walk/drive); the percentage of members giving support who live nearby (5-minute walk/drive) – a measure of network dispersion; the number of frequent contacts (members in contact at least weekly, including by phone, email or social media); number of cohabitants; and whether the network included a spouse or partner.

#### Social Network characteristics

Measures of each patient's social network, and social networking, included the number of different relationship types present in the network (out of 10 types: immediate family (including spouse), extended family, friends, neighbours (if not classified a friend), groups, health professionals, other professionals, work relationships, pets, other ); network density (number of network members pairs who know each other out of all possible pairs); the amount of support given by the participant to others in the past month (a count out of seven kinds of possible support); and – as measures of access to network resources and social capital - a score on a social resources measure (resource generator [44]); and extent of wider (ie beyond the family) social involvement (number attended out of 14 different types of group or organisation). We did not use overall network size (total number of members) as this demonstrated multicolinearity with other variables in the study (see below).

To quantify the contribution to illness management made by the members of each network, we devised a Likert scale questionnaire to assess perceived contribution (from 1 = not at all to 5 = a lot) of each member to each of 13 aspects of work. Each member's ratings were then summed across the items in three separate domains of emotional work, illness work and everyday work and rescaled from 0 = does not help at all in any aspect, to 10 =  helps a lot in all aspects, then summed across members to obtain overall scores for each network (for full details see Vassilev et al [20], [21]). However, scores on the three work domains correlated one with another above 0.6 and in analysis we experienced multicolinearity problems between these measures. We therefore decided to retain the illness work domain as the most relevant for the focus of this paper, and removed the others.

A wide variety of constructed variables can be derived from social network data, therefore to avoid any potential for ‘fishing’ for significant results all of our constructed measures were specified in full and computed prior to conducting any analysis of relationships to outcomes. It was not possible to compute in-sample reliabilities for the network measures, but previous studies of adult support networks using similar methods have generally reported reliabilities for network-level measures substantially higher than 0.8 [45], [46].

### Measures of Network Change

We constructed two measures of the extent to which each patient's network had changed across the 12-month period. The first was a binary measure (yes/no) indicating whether or not a network had lost one or more members considered important (positioned in either the central or middle circle of the network) by the patient at time period 1. The second measure was the sum total across all network members of all the work (of any type) done at Time 1 by people no longer in the network at Time 2. Both of these measures are indicative of loss of either people or work from the networks. Ideally measures of network gain would also have been constructed, but the different method used to collect follow-up data did not permit this.

### Analysis Methods

The focus of the analysis was on the relationships between health-related outcomes and characteristics of the social networks. Socio-demographic factors were controlled for as a “block” of variables in the analysis, so as to remove potential confounding with the network measures. We also controlled separately for the measures of disease burden for the same reason. We excluded the measures of network size, emotional and practical work due to multicolinearity; all other explanatory factors in the analysis had variance inflation factors (VIFs) no higher than 2.3 [47].

We conducted two sets of analyses. The first explored relationships between personal network characteristics and scores on the outcome measures at Time 1; the second analysis repeated this but using the change in each outcome from Time 1 to Time 2 as the dependent variable. Change scores can be subject to ‘mathematical coupling’ and regression to the mean, which in randomised studies are controlled by including Time 1 scores as a covariate in analysis. However, in observational studies when subgroup means differ at Time 1 this approach introduces bias into the estimates of subgroup differences in mean change [48]. Since we expected subgroup differences at Time 1 (eg, outcomes may differ by gender) we did not control for scores at Time 1. This produces unbiased estimates of subgroup differences, although standard errors may be somewhat inflated.

For each outcome we ran three analysis models using multivariate regression. Model 1 examined the relationship between each outcome and each explanatory variable in turn, controlling for patient demographics (age, gender, ethnicity, residential deprivation, occupational class, highest qualification and income). Model 2 added the measures of disease burden (number of long-term conditions and main condition (CHD, diabetes, or both)), to determine if controlling for this changed the strength of association. Model 3 repeated model 2, but included all network variables together in a backwards stepwise procedure to identify the set of factors most predictive of each outcome.

In the case of service costs, the distribution was highly skewed with two exceptionally large values (more than twice the next largest). To account for this, we followed a recommendation to apply standard regression using a bootstrap estimate of standard error, and to repeat the analysis with and without the extreme values [49]. To investigate whether our measure of informal care (the illness work performed by the network) substituted for or complemented levels of formal care (service costs), we applied instrumental variable analysis using a two-stage least squares regression model [50], and performed tests for the strength of the instruments, overidentification and endogeneity [25]. The instrumental variables for illness work were numbers of male and female children, assumed to affect amount of informal care but not directly the use of formal care [25], [26]. Female children generally provide more care than male children and we therefore both as two joint instruments.

To assess sensitivity of change scores to missing outcome values at follow-up we used multiple regression to impute missing values using the full set of variables at baseline and repeated the analysis using this dataset. We report on results that changed statistical significance under sensitivity. All analyses were conducted in Stata v12. Many of the outcomes displayed non-normal distributions, therefore for significance testing we used the Huber-White estimator of variance, which is known to be robust against departures from normality [51]. An alpha-level of 5% was used throughout to designate a statistical significant result.

## Results

Rates of missing data at Time 1 were low: for most variables zero or well under 5%. We used a combination of mean and regression imputation to impute missing Time 1 values (for full details see Vassilev et al [20].

The sample was around two-thirds male (64%), predominantly white (86%), and with a mean age of 65 years (Table 1; additional demographic information is given in Table S1 in File S1). Nearly one-fifth (19%) had diabetes as their main condition, 40% had CHD and 40% had both diabetes and CHD; just over 50% had three or more long-term conditions. Over half the participants were married (55%, n = 165), almost half were retired (49%, n = 148) and 43% had no qualifications beyond basic school level. We had targeted a deprived population and this is reflected in the fact that 52% of participants lived in the 20% most deprived local areas in England [40].

**Table 1:** Descriptive statistics of the sample.

**Patient characteristics**		**N (%)**
Gender	Male	193 (64.3%)
	Female	107 (35.7%)
Age (mean (SD) range)		65.3 (12.6) 20–93
Main condition(s)	Diabetes	58 (19.3%)
	Chronic Heart Disease	120 (40.0%)
	Both conditions	122 (40.7%)
Number of long-term conditions^1^	1	49 (16.3%)
	2	93 (31.0%)
	3	83 (27.7%)
	4	43 (14.3%)
	5 or more	32 (10.7%)
Area Index of multiple deprivation (mean (SD) range)		37.5 (19.3) 5.3–78.1
**Network member characteristics**		
Number of children nearby^1^ (cohabiting or short walk/drive)	None	118 (39.3)
	1	63 (21.0%)
	2	59 (19.7%)
	3	38 (12.7%)
	4 or more	22 (7.3%)
% of network members nearby (mean (SD) range)		36.5% (22.2) 0–100
Number of frequent contacts (daily or weekly) (mean (SD) range)		4.8 (3.0) 0–18
Number of cohabitants	None	76 (25.3%)
	One	132 (44%)
	Two or more	92 (30.7%)
Network includes spouse/partner	No	123 (41%)
	Yes	177 (59%)
**Social network characteristics**		
Network density (mean (SD) range)		0.49 (0.18) 0.11–1.0
Mix of agents in network (mean (SD) range)		3.4 (1.3) 0–7
Number of community/voluntary groups attended in last month^1^	None	130 (43.3%)
	1	82 (27.3%)
	2	42 (14.0%)
	3	28 (9.3%)
	4 or more	18 (6.0%)
Help given to others^1^	None	142 (47.3%)
	One type of help	76 (25.3%)
	Two or more types	82 (27.3%)
Social resources (mean (SD) range)		39.1 (23.8) 0–100
Illness management work from network (mean (SD) range)		18.6 (11.3) 0–57.1
Emotional work from network (mean (SD) range)		30.3 (20.3) 0–128.1
Everyday work from network (mean (SD) range)		12.4 (9.1) 0–46.7
**Measures of network change (from Time 1 to Time 2)**		
Lost one or more important network members	No	219 (88.3%)
	Yes	29 (11.7%)
Total amount of work lost from the network (mean (SD) range)		3.2 (9.48) 0–80.5

1 Used as a continuous variable in regression analysis.

Summary statistics for all the outcome measures at Time 1 and Time 2 appear in Table 2. Fifty-two patients (17%) did not return any self-report measures at Time 2.

**Table 2:** Summary statistics for outcome measures.

Explanatory variable	Time 1 [N Mean (SD)]	Time 2 [N Mean (SD)]	Change [N Mean (SD)]	Correlation between Time 1 and Time 2 scores [N rho]
**Health-related outcomes**				
Self-management (HEIQ; scale 1 to 4)	300 2.98 (0.46)	248 3.08 (0.45)	248 0.08 (0.44)	248 0.56***
Healthy behaviours (SDSCA; scale 0 to 7)	300 3.61 (1.13)	248 3.61 (1.11)	248–0.03 (3.04)	248 0.69***
Physical health (SF12)	300 50.0 (10.0)	248 49.68 (10.27)	248–0.91 (6.73)	248 0.78***
Emotional well-being (Scale 0 to 100)	300 68.72 (23.95)	248 68.07 (23.76)	248–2.15 (17.31)	248 0.73***
**Health economics outcomes**				
Health service costs (in £'s)	300 £640 (£1746)	248 £656 (£1809)	248 £45.1 (£2451)	248 0.06 ns
QALYs over the 12 months (in days)	247 239.1 (51.8)		NA	NA

***p< = 0.001; ns =  not significant (p>0.05); NA: not applicable.

### Health Outcomes at Time 1

This paper focuses on relationships between health outcomes and social network characteristics, but for completeness a multivariate analyses of associations with sociodemographic and disease factors is summarised in Table S2 in File S1. The strongest predictors of all four health outcomes were one or both disease burden measures (main long-term condition(s) and total number of long-term conditions), with a smaller impact of income, age and residential deprivation on some health outcomes. This demonstrates the substantial impact that disease burden had on all aspects of health in these patients and the importance of controlling for this in analysis.

Network member characteristics showed very few significant relationships with any health outcome (Tables 3 and 4). The main exception was number of proximate children, with negative coefficients indicating that patients with more children living nearby on average reported a lower level of healthy behaviours (Table 3) and poorer physical health (Table 4). A significant positive relationship between the presence of a partner in the network and emotional well-being (Table 4) ceased to be significant once disease burden was taken into account.

**Table 3:** Summary of regression analyses of outcomes at Time 1: Self-management outcomes.

Explanatory variable^1^	Self-management			Healthy behaviours		
	Model 1	Model 2	Model3	Model 1	Model 2	Model3
	Coeff (SE)	Coeff (SE)	Coeff (SE)	Coeff (SE)	Coeff (SE)	Coeff (SE)
Member characteristics						
Number of proximate children				−.16** (.06)		−.14* (.06)
**Social network measures**						
Density		−.28* (.14)				
Social involvements	.060** (.022)	.059** (.022)	.053* (.021)			
Help given to others	.064* (.030)	.068* (.029)	.059* (.029)			
Illness work		.004* (.002)	.005* (.002)			.014* (.007)

*p< = 0.05;

**p<0.01;

***p< = 0.001.

1 Variables with no significant relationships to any outcome are not shown.

Model 1 = individual network characteristics controlled for patient demographic variables.

Model 2 = individual network characteristics controlled for patient demographics and disease burden.

Model 3 = network characteristics controlled for demographics, disease burden and one-another.

**Table 4:** Summary of regression analyses of outcomes at Time 1: Health status outcomes.

Explanatory variable^1^	Physical health			Emotional well-being		
	Model 1	Model 2	Model3	Model 1	Model 2	Model3
	Coeff (SE)	Coeff (SE)	Coeff (SE)	Coeff (SE)	Coeff (SE)	Coeff (SE)
Member characteristics						
Number of proximate children	−1.26** (.42)		−.71* (.36)			
Partner/spouse in network				6.23* (3.01)		
**Social network measures**						
Mix of agents				2.43* (1.11)	2.53* (1.05)	
Social involvements	1.35*** (.42)	1.38*** (.37)	1.25** (.37)	2.90** (1.07)	3.04** (1.06)	3.06** (1.05)
Help given to others		1.43** (.57)	1.19* (.56)			
Illness work	−.15*** (.05)				.32** (.12)	.32** (.11)

*p< = 0.05;

**p<0.01;

***p< = 0.001.

1 Variables with no significant relationships to any outcome are not shown.

Model 1 = individual network characteristics controlled for patient demographic variables.

Model 2 = individual network characteristics controlled for patient demographics and disease burden.

Model 3 = network characteristics controlled for demographics, disease burden and one-another.

A larger number of relationships were observed between health outcomes and the social network characteristics. Patient ability to self-manage displayed the largest number of associations, with (depending upon the model) a less dense network, greater social involvement, giving more help to others, and receiving more illness work through the network (Table 3). Conversely, levels of healthy behaviours showed only one relationship, with higher levels of illness work, but only after disease burden and proximate children had entered the model (Table 3).

**Figure 1:**
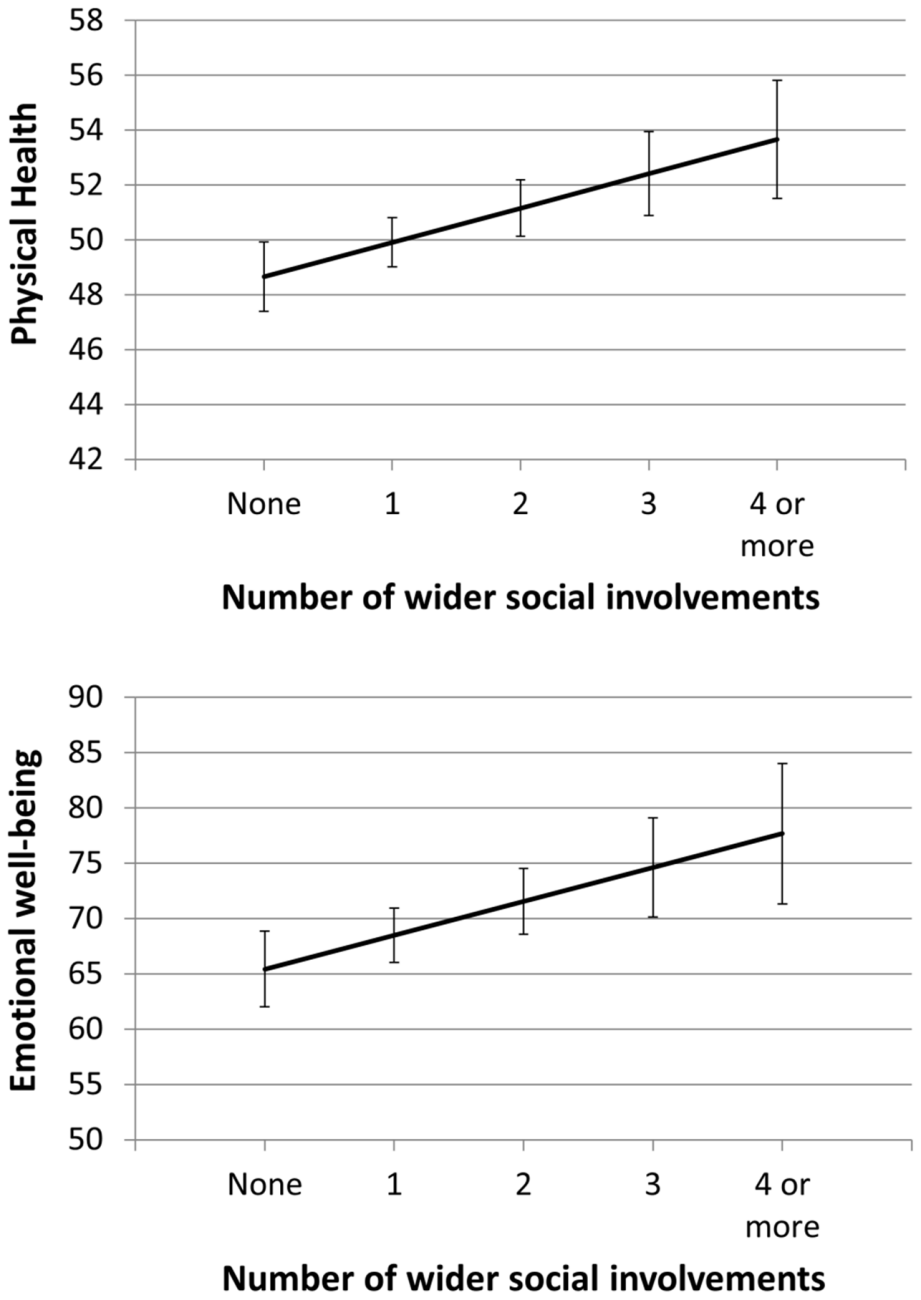
Modelled relationships (mean score and 95% confidence interval) between physical health and number of social involvements, and emotional well-being and number of social involvements, controlled for patient sociodemographics, disease burden and other significant social network characteristics (Model 3).

**Figure 2:**
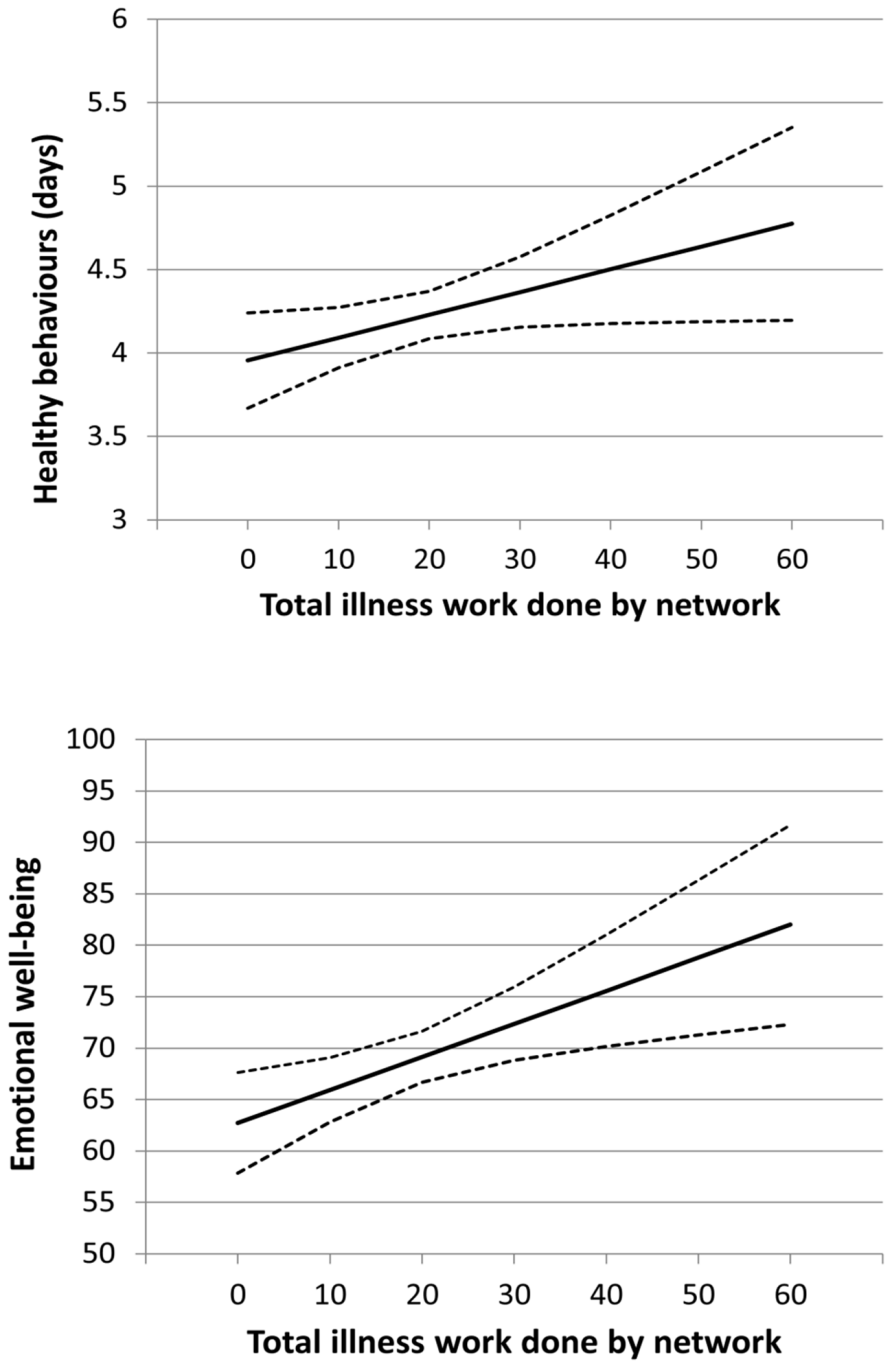
Modelled relationships (mean score and 95% confidence interval) between health behaviour and illness work done by the network, and emotional well-being and illness work done by the network, controlled for patient sociodemographics, disease burden and other significant social network characteristics (Model 3).

Across the set of all four health outcomes, three social network factors displayed relationships with more than one outcome. Greater social involvement was significantly related to better self-management ability, better physical health and greater emotional well-being under all three analysis models (Figure 1). Help given to others was also positively related to self-management (all models) and physical health (after controlling for disease burden). Illness work demonstrated significant relationships with all four outcomes, though with differing patterns. Greater amounts of Illness work were positively related to increased self-management, healthy behaviours and emotional well-being, but only after controlling for disease burden: that is to say, for people experiencing similar levels of disease burden, those receiving more illness work reported more ability to self-manage and better physical and emotional health (Figure 2). Illness work was negatively related to physical health prior to adjustment for disease burden - indicating that illness work levels were higher for those in poorer health - but not related afterwards, which suggests that the illness work provided by a network was largely proportionate to the illness burden experienced by the patient.

### Change in Health Outcomes

Sample mean scores for all outcome measures changed little over the 12 months (Table 2). However, there were substantial amounts of change at the individual level in self-management (r = 0.56; p<0.001) and service costs (r = 0.06; p = 0.39), though physical health (r = 0.78; p<0.001), emotional well-being (r = 0.73; p<0.001) and healthy behaviours (r = 0.69; p<0.001) showed far less individual change.

The ability of the explanatory variables to account for changes in patient-reported outcomes over the 12 months of the study was quite limited (Tables 5 and 6). We found no significant predictors of change in self-management ability, but physical health status was found to have declined for those with a partner in their network. There was a positive association between change in healthy behaviours and number of social involvements: closer inspection revealed a decline in healthy behaviours for patients with no social involvements (mean drop of −1.3) but a small increase for those with involvements (mean of 0.62). Healthy behaviours also decreased for those who had lost illness management help from their network. Similarly, emotional well-being dropped over the 12 months for patients who had lost important members from their network.

**Table 5:** Summary of regression analyses of changes in outcomes: Self-management outcomes.

Explanatory variable^1^	Self-management			Healthy behaviours		
	Model 1	Model 2	Model3	Model 1	Model 2	Model3
	Coeff (SE)	Coeff (SE)	Coeff (SE)	Coeff (SE)	Coeff (SE)	Coeff (SE)
Member characteristics						
Partner/spouse in network	−.12 (.06)$					
**Social network measures**						
Social involvements				.16** (.06)	.15** (.06)	.13* (.06)
**Network change**						
Loss of work from network				−.021* (.009)	−.020* (.009)	−.018* (.009)

*p< = 0.05;

**p<0.01;

***p< = 0.001.

$ Statistically significant (p<0.05) under sensitivity analysis.

1 Variables with no significant relationships to any outcome are not shown.

Model 1 = individual network characteristics controlled for patient demographic variables.

Model 2 = individual network characteristics controlled for patient demographics and disease burden.

Model 3 = network characteristics controlled for demographics, disease burden and one-another.

**Table 6:** Summary of regression analyses of changes in outcomes: Health status outcomes.

Explanatory variable^1^	Physical health			Emotional well-being		
	Model 1	Model 2	Model3	Model 1	Model 2	Model3
	Coeff (SE)	Coeff (SE)	Coeff (SE)	Coeff (SE)	Coeff (SE)	Coeff (SE)
Member characteristics						
Number of frequent contactors					.67 (.39)$	
Partner/spouse in network	−2.14* (.94)	−1.86* (.94)	−1.86* (.94)			
**Network change**						
Loss of key network members				−6.69* (3.23)	−6.45* (3.21)	−6.45* (3.21)

*p< = 0.05;

**p<0.01;

***p< = 0.001.

$ Statistically significant (p<0.05) under sensitivity analysis.

1 Variables with no significant relationships to any outcome are not shown.

Model 1 = individual network characteristics controlled for patient demographic variables.

Model 2 = individual network characteristics controlled for patient demographics and disease burden.

Model 3 = network characteristics controlled for demographics, disease burden and one-another.

### Health Economics Outcomes

Patient QALYs for the 12 months of the study were found to be higher (p<0.05) for patients with more social involvements, under all analysis models (Table 7). Significant negative relationships were found with levels of illness work and with numbers of proximate children. However, these latter relationships were no longer statistically significant after controlling for disease burden.

**Table 7:** Summary of regression analyses of health economics outcomes.

Explanatory variable^1^	QALYs (n = 247, expressed in days)			Health service costs (Time 1, £'s)		
	Model 1	Model 2	Model3	Model 1	Model 2	Model3
	Coeff (SE)	Coeff (SE)	Coeff (SE)	Coeff (SE)	Coeff (SE)	Coeff (SE)
Member characteristics						
Number of proximate children	−7.59*** (2.31)					
**Social network measures**						
Social involvements	5.80* (2.61)	4.92* (2.34)	4.92* (2.34)			
Illness work	−.69** (.27)			−19.53** (6.66)	−21.92** (8.61)	−21.92** (8.61)

*p< = 0.05;

**p<0.01;

***p< = 0.001.

1 Variables with no significant relationships to any outcome are not shown.

Model 1 = individual network characteristics controlled for patient demographic variables.

Model 2 = individual network characteristics controlled for patient demographics and disease burden.

Model 3 = network characteristics controlled for demographics, disease burden and one-another.

Health service costs at Time 1 were significantly (p<0.01) reduced for patients receiving greater levels of illness work through their networks, both with and without adjustment for disease burden. No other network factor showed any relationship to service costs. We found no significant predictors of change in service costs between Time 1 and Time 2. These results did not change when we excluded the two cases with extreme costs. Mean 6-month service costs for patients in the upper third of the illness work distribution (£362; 95% CI £239 to £486) were less than half those for patients in the lower third (£766; 95% CI £502 to £1030). Examination of the different components of costs revealed that the largest part of the cost reduction was due to fewer overnight stays in hospital for patients receiving high levels of illness work through their network, with an average stay in hospital of 0.3 days compared to 1.1 for patients receiving low levels of illness work.

For the instrumental variable analysis, the test of the joint significance of the instruments for illness work (numbers of male and female children) indicated that these were sufficiently strong (F(2,297) = 11.26; p<0.001) and also passed the overidentification test (non-significant test of overidentifying restrictions: Model 1 Chi-square = 2.6, p = 0.11; Model 2 Chi-square = 2.4, p = 0.12). The analysis did not suggest that illness work is endogenous (non-significant Hausman exogeneity test: Model 1 F(1,290) = 0.001, p = 0.97; Model 2 F(1,287) = 0.21, p = 0.65), and although the regression coefficients were non-significant (Model 1 −£18.57, p = 0.47; Model 2 −£34.45, p = 0.23), they were of the same direction and size as for the non-instrumented analysis. Thus the preferred solution is with illness work exogenous to (i.e. not influenced by) service costs.

**Figure 3:**
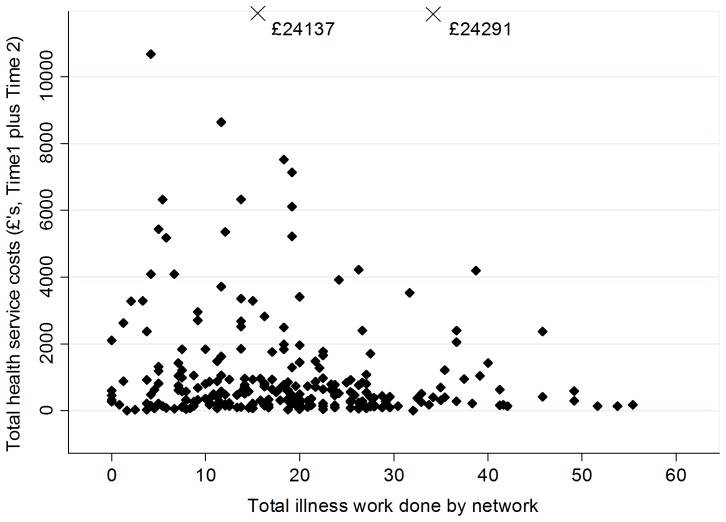
Total service costs (Time 1 plus Time 2) by amount of illness work done by the network. X  =  outlier value.

In view of the potential importance of a relationship between illness work and costs, to increase our power for testing this association we analysed the total service costs across Time1 and Time 2 combined. The relationship remained significant both with (beta = −£29.80; p = 0.042) and without (beta = −£25.49; p = 0.007) inclusion of the outliers. The plot of combined service costs against illness work (Figure 3) suggests a narrowing of the range in costs as the level of illness work increases.

### Results of Sensitivity Analysis

Sensitivity analysis with missing values replaced by imputed scores from multiple regression, produced very little change, with just two results changing from being of borderline significance (p<0.1) to being significant (p<0.05) (Tables 6 and 7). No associations changed from significance to non-significance.

## Discussion

### Summary of Findings

Social networks have been seen as playing a potentially important but relatively unspecified role in providing self-management support for long term conditions [5], [21] and this study was motivated by the idea that these provide people with long term conditions access to relationships and resources which can support them in managing their condition(s). This study has demonstrated associations between the properties of an individual's social network and positive outcomes for health. Of most note are three findings which indicate that: (1) social involvement with wider resources (e.g. community groups) supports personal self-management and physical and mental well-being; (2) that the support work undertaken by personal networks expands in accordance with health needs and that this helps people cope practically and emotionally with their condition but does not impact on health per se; and (3) that network support substitutes for formal care and can produce substantial savings in traditional health service utilisation costs.

With regard to social involvement, being connected to voluntary and community groups was related to key dimensions of self-management (self-monitoring and skill and technique acquisition, as measured by the HEIQ), as well as to better physical health and emotional well-being. Significantly, social involvement was also associated with the maintenance of healthy behaviours over time, with these behaviours declining in patients who had no links to community groups or organisations. Although this analysis does not reveal the precise nature and directions of these relationships, the findings do suggest that social involvement may impact on personal capabilities to self-manage, possibly through the provision of sources for information but more likely as a means of keeping the individual engaged and active in normal life [52]. The association of help given to others with better self-management and physical health scores highlights the importance of activities which are reciprocal as well as altruistic in promoting good self-management. The gaining of independence and autonomy through social networks outside of the immediate domestic environment has been highlighted previously [19]; in this respect links to groups which allow for social involvement may perform a similar function for people with a chronic condition.

We found that a higher amount of illness work by network members was associated with poorer physical health and reduced QALYs. We also found that people with poorer health or less healthy behaviours tended to have more children living nearby. A plausible interpretation for these relationships is that networks respond to poorer health by providing more support. The dominant factor here is the network responding to the patient's health status such that family and network members may ‘rally around’ patients in poorer health by increasing levels of support, including moving closer in order to do so. These findings accord with the “positive care law” described by Shaw and Dorling[22] by which the provision of informal care is positively related to need. However, whereas Shaw and Dorling reported a relationship at the area level, potentially subject to the ecological fallacy [53], our results demonstrate that the law does indeed operate at the level of individual patients. When we controlled for degree of illness burden in our models further relationships emerged: for patients at similar levels of disease burden those receiving more illness work through their network did not show better physical health, but did show greater ability to self-manage, better emotional health and more healthy behaviours. Thus although greater network support did not improve physical health per se, it did improve patients' ability to cope with their condition(s), both practically and emotionally. Our definition of what constitutes illness work goes well beyond just the kinds of activities undertaken by health professionals, to include illness related activities by network members in everyday settings and in interfacing between the patient and formal services. Indeed, we have previously shown that health professionals provide only a small fraction of the totality of all illness work [20]. Partners and close family members make the highest contributions, but importantly there is also evidence for inputs from a wide range of other relationships including those considered to be ‘weak ties’ [54], [55]. Independently of relationship type, network members who are female, live nearby, or contact more frequently, provide the highest levels of illness work, as do denser networks (ie where more members know each other) [20]. In the present analysis however, network member characteristics displayed far fewer associations with health outcomes than did the sum total of illness work across the full network. This suggests that the totality of support is more pertinent to patient outcomes than the specific individuals contributing that work, and hints at a high degree of substitutability between members. In addition, although for empirical reasons of analysis we have focused on illness-specific work in this paper, the dividing lines between this and practical and emotional support - particularly the latter - were very blurred, with the same network members often central to all three.

Our third main finding was of associations between network characteristics and health economics outcomes. Greater social involvement was associated with increased quality adjusted life years over a 12 month period. However, of potentially more importance, was the relationship between levels of illness work provided by the members of a patient's network and the cost demands a patient makes on the health service. In general, health service costs for patients receiving the highest levels of illness work were nearly half the costs for patients receiving the lowest levels, and most of the cost saving was due to a reduction in hospital bed days. A possible mechanism here is that patients receiving higher levels of network support were more able to be looked after at home and so discharged earlier. This finding concurs with that of Van Houtven and Norton for the USA [25] but is at odds with what Bolin found for Europe [26]. Our instrumental variable analysis also concurred with Van Houtven and Norton in finding that informal care substitutes for, rather than complements, formal care. This result clearly needs validation in further studies but if correct, the implication is that considerable health service cost saving could accrue from investing in increasing the illness support people receive from their personal networks.

We found only a few significant relationships between social network measures at Time 1 and change in patient outcomes across the subsequent 12 months. However, physical and emotional well-being remained fairly stable over this period and it may be that this was too short a time for detecting many long-term effects; although notwithstanding this we did find that loss of members from a network led to reductions in healthy behaviours and well-being over time. Unfortunately we were unable to examine the effects of changes in the make-up or structure of the networks other than loss of members. For researchers planning future studies we therefore recommend collecting full network data at baseline and follow-up in order to explore network dynamics in more depth. Also, some factors identified by our analysis as playing a key role would benefit from being assessed in a more nuanced way, in particular illness burden and social involvement. Ideally, important network changes should be time-stamped and outcomes collected at more frequent intervals and over a longer period, so as to allow a more refined causal analysis of the two-way dynamics at work. Finally, we would advise the inclusion of a group of patients with similar demographic backgrounds but without long-term conditions, to increase variability across the sample.

### Limitations

The response rate to our invitation letters was low (15%), which is typical of surveys that target disadvantaged populations. Respondents generally had low levels of formal educational attainment and lived in very deprived areas. We lacked data with which to make comparisons with non-respondents and cannot say if social networks and their influence on health outcomes may have differed between these groups. The directions of effect assumed within the regression models may be at odds with the actual directions that were operating for some variables. We found strong indications that some important network characteristics, most notably the illness work done and the geographical closeness of children, were responding to patient health needs, at least as much as they impacted on outcomes in return, thus caution is required when interpreting direction of effect. All participants had at least one chronic condition and lived in areas of high deprivation, which is likely to have restricted the variation both in outcomes and in social network measures, compared to a general population including healthier and more affluent individuals. The effect would have been to reduce our ability to detect relationships and to reduce the strength of the relationships we did find. We conducted a large number of statistical tests but used an alpha level of 5% throughout: exploratory studies need to balance the risks of both Type 1 (false positive) and Type 2 (false negative) errors and we did not want to miss potentially important findings by setting too high an alpha level [56]. However, this does mean that some of the relationships we report may be spurious and all our results need to be validated in confirmatory studies. Our method for collecting network data at baseline proved very time and resource intensive which is why we changed the approach at follow-up. However, this considerably limited our ability to examine network dynamics.

### Conclusion

This study has made some progress towards a better understanding of the interplay between the social networks of people with long-term conditions, their health care needs, and their abilities to cope with their conditions. In particular, it is evident that social networks are adaptable and responsive to levels of health need, and that the overall support provided by the network is more salient than the particulars of the individual members, whilst involvement in social organisations and reciprocal or altruistic activities provides additional, independent, health benefits. Support for self-management is therefore more meaningfully construed as a collective and networked phenomena, rather than as a set of individualised actions and behaviour. This study shows the need for a greater focus on harnessing and sustaining the capacity of networks and the importance of social involvement with community groups and resources as a means of achieving desirable policy outcomes and a more cost-effective way of supporting long term illness management.

## Supporting Information

File S1 This file contains Table S1 and Table S2. Table S1, Additional descriptive statistics of the patient sample. Table S2, Summary of regression analysis of outcomes at Time 1 by patient characteristics.
(DOCX)
