# Supplementary material for: The Contribution of Social Networks to the Health and Self-Management of Patients with Long-Term Conditions: A Longitudinal Study
Source: PLoS One. 2014 Jun 2;9(6):e98340. doi: 10.1371/journal.pone.0098340 (PMC4041782; doi:10.1371/journal.pone.0098340)
Supplement: File S1 — This file contains Table S1 and Table S2. Table S1, Additional descriptive statistics of the patient sample. Table S2, Summary of regression analysis of outcomes at Time 1 by patient characteristics. (DOCX) [file pone.0098340.s001.docx]

Table S1: Additional descriptive statistics of the patient sample

| **Patient characteristics** | | **N (%)** |
| --- | --- | --- |
| Ethnicity | White | 259 (86.3%) |
|  | Non-white | 41 (13.7%) |
| Occupational class^1^ | 1 (highest) | 30 (10.0%) |
|  | 2 | 53 (17.7%) |
|  | 3 | 40 (13.3%) |
|  | 4 | 47 (15.7%) |
|  | 5 | 42 (14.0%) |
|  | 6 | 35 (11.7%) |
|  | 7 | 53 (17.7%) |
| Highest qualification^1^ | No qualification | 75 (25.0%) |
|  | School qualifications (eg ‘O’ level, GCSE) | 54 (18.0%) |
|  | College qualifications (eg ‘A’ level) | 59 (19.7%) |
|  | Vocational (eg NVQ, HNC, HND) | 39 (13.0%) |
|  | Degree or professional qualification | 54 (18.0%) |
|  | Higher degree (eg MA, PhD) | 19 (6.3%) |
| Income^1^ | Up to £5,199 | 17 (5.7%) |
|  | £5,200-£10,399 | 82 (27.3%) |
|  | £10,400-£15,599 | 78 (26.0%) |
|  | £15,600-£20,799 | 49 (16.3%) |
|  | £20,800-£25,999 | 25 (8.3%) |
|  | £26,000-£31,999 | 19 (6.3%) |
|  | £32,000-£51,999 | 21 (7.0%) |
|  | £52,000 or more | 9 (3.0%) |

^1^Used as a continuous variable in regression analysis

Table S2: Summary of regression analysis of outcomes at Time 1 by patient characteristics

| **Explanatory variable** | | **Self-management** | **Healthy behaviours** | **Physical health** | **Emotional well-being** | **QALYs** | **Service costs^3^** |
| --- | --- | --- | --- | --- | --- | --- | --- |
|  |  | Coeff (SE) | Coeff (SE) | Coeff (SE) | Coeff (SE) | Coeff (SE) | Coeff (SE) |
| **Sociodemographic characteristics** | | | | | | | |
| Age | |  | .013* (.006) |  | .42*** (.13) | .71** (.24) |  |
| Gender (Female) | | .12* (.057) | .34* (.15) |  |  |  |  |
| Ethnicity (non-white) | |  | .51* (.20) |  |  |  |  |
| Deprivation (IMD) | |  |  | -.053* (.027) |  | -.35* (.15) |  |
| Occupational class | |  |  |  |  |  |  |
| Qualifications | |  |  |  |  |  |  |
| Income | |  |  | 1.11*** (.29) | 1.75* (.87) | 6.02*** (1.67) |  |
| **Disease burden** | | | | | | | |
| N of conditions | | -.087*** (.026) | -.26*** (.067) | -4.57*** (.44) | -8.69*** (1.28) | -23.1*** (2.35) |  |
| Main conditions^12^ | CHD |  | -.16*** (.22) | -1.26* (1.35) | -.75** (3.50) | -16.9*** (7.78) |  |
|  | CHD + diabetes |  | .58 (.23) | 3.29 (1.44) | 10.25 (3.71) | 6.53 (8.48) |  |

*p<=0.05; **p<0.01; ***p<=0.001

^1^Compared to diabetes

^2^Significance refers to joint test across all three levels

^3^No significant relationship found with any explanatory variable
